# Training surgical skills on hip arthroscopy by simulation: a survey on surgeon’s perspectives

**DOI:** 10.1007/s11548-022-02708-x

**Published:** 2022-07-13

**Authors:** Bohong Cai, Shengfeng Duan, Jiahui Yi, Wei Huang, Boon Huat Bay, Chunbao Li, Cheng Chen

**Affiliations:** 1 Department of Industrial and Product Design, School of Design, Sichuan Fine Arts Institute, Chongqing, China; 2 Department of Orthopedics, The First Affiliated Hospital of Chongqing Medical University, Chongqing, 400016 China; 3 Department of Anatomy, Yong Loo Lin School of Medicine, National University of Singapore, Singapore, Singapore; 4 Department of Orthopedics, The Fourth Medical Center of Chinese PLA General Hospital, Beijing, 100048 China

**Keywords:** Hip arthroscopy, Surgical training, Medical simulation, Surgeon

## Abstract

**Purpose:**

The purpose of this study is to investigate the importance of general and specific surgical skills for hip arthroscopy from the perspective of surgeons in China. Concurrently, we intend to identify the preferred type of simulation that would facilitate competency of surgical trainees in performing arthroscopy and reinforce their preparation for carrying out the actual surgical procedure.

**Methods:**

An online survey comprising 42 questions was developed by experts in hip arthroscopy and sent to 3 online communities whose members are arthroscopic surgeons in China. The responses collected were based on a 5-point Likert scale, with an open-ended comment section. Data were analyzed using one-way AVOVA and post hoc Tukey’s test.

**Results:**

A total of 159 valid responses from 66 junior specialist surgeons, 68 consultant surgeons, and 25 senior consultant surgeons (from 130 institutions in 27 out of 34 provincial administrative districts in China) were collected. Cognitive ability was identified as the overall most important attribute for hip arthroscopic trainees to possess, while skills relevant to the treatment of femoroacetabular impingement (FAI) were considered as the most important specific skills by the surgeons surveyed. In addition, simulation using cadaveric specimens was considered the most favorable method for surgeons to practice their surgical skills.

**Conclusion:**

In designing a training program for hip arthroscopy, it is essential to incorporate features that evaluate cognitive skills. It would be helpful for trainees to specifically practice skills that are often used in the treatment of some very common diseases of the hip joint, such as FAI. Using high-fidelity physical models for simulation to train skills of hip arthroscopy could be an ideal alternative and effective way to overcome problems arising from the lack of accessibility to cadaveric specimens.

**Supplementary Information:**

The online version contains supplementary material available at 10.1007/s11548-022-02708-x.

## Introduction

Hip arthroscopy is one of the most important hallmarks of arthroscopic techniques. This surgical technique has been used for the treatment of labral tears, chondral defects, ligamentum teres lesions, and femoroacetabular impingement (FAI) [1, 2]. As a minimal invasive surgical technique compared to open surgery, hip arthroscopy is often challenging due to difficulties such as reduced tactile feedback, restricted field of vision, limited degrees of freedom of instrument, and the requirement of integrating two-dimensional (2D) information projected on screens and mental reconstruction into useful three-dimensional (3D) spatial information [3–5]. Furthermore, there are additional concerns such as safe access to the anatomical location, as well as time constraints for the surgery that need to be taken into consideration as a whole by hip arthroscopic surgeons, in order to avoid or reduce the risk of iatrogenic injury [5]. The challenges and limitations of hip arthroscopy increase the learning difficulty of this specialized surgical procedure [6].

However, the implementation of simulation-based training has shown great potential in enhancing hip arthroscopic training [7, 8], and different types of simulators have already been used in arthroscopic training [8–12]. Simulation-based training provides a risk free and conducive learning environment for trainees to hone their surgical skills [12]. Previous studies have revealed that simulation is an effective method for learning technical skills, with the skills acquired being transferable to clinical settings [12, 13]. Moreover, simulation-based training also shortens the learning curve and enhances intraoperative learning capacity [14]. Hence, reducing the training duration required for surgical trainees to acquire proficiency in hip arthroscopy [15, 16]. Before the advent of simulation-based training, it was common for novice surgeons to spend years in the operating room, practicing their surgical skills before they can achieve an optimal level of skills.

Despite mounting evidence that support the effectiveness of simulation in surgical training, the orthopedic community has still not fully embraced this advanced educational method [17]. Conceivably, this is because implementing simulation-based training is often a complex organizational effort that involves many people at different stages of implementation and necessitates adaptation depending on the local scenarios [17]. As highlighted by Sutherland et al. [18], the three main factors affecting simulation-based training are the simulator, educator, and curriculum. Hence, it is important to elucidate the relationship between these factors, and doing so would aid in improving the efficiency of the simulator, and augmenting the strategy for curriculum design.

Earlier research studies conducted by Safir et al. [19] and Hui et al. [20] have revealed a list of demanding arthroscopy skills that should be trained in simulation settings. Based on their research findings, the most important skills for arthroscopic surgery from the perspectives of surgeons and residents were related to identification of anatomical structures, and the preferred type of simulation was the use of cadaveric specimens. These findings are useful as a basis for developing a practical framework for simulation-based training program for arthroscopic surgeons. However, the research focused on surgeons and residents in the Canadian orthopedic community (Canadian Orthopedic Association and Canadian Orthopedic Resident Association) and only encompass knee related arthroscopic skills, but not other skills required to operate on other joints such as the hip. Moreover, it is known that work culture can vary drastically depending on customs, lifestyle, and the region concerned [21]. Therefore, to develop an appropriate training program, it is necessary for medical professionals to investigate the characteristics of a specific clinical procedure which is based on their own local community [22, 23].

In this study, we investigated the importance of general and specific surgical skills for hip arthroscopy. Concurrently, we also explored which simulation method is most effective for arthroscopic training from the perspective of experienced surgeons, as well as how simulation can affect surgeons’ learning behavior and performance in the operating room.

## Method

The invitation to participate in the online survey was published in three social media groups organized by Chinese Medical Association and exclusively used by specialists of Lower Limb Sports Medicine. The invitation included a brief introduction that described the purpose of the study and an internet link to access the online survey. The online survey was developed using the Website www.wenjuan.com and had 42 questions adapted from the work of Safir et al. [19], which were modified by 3 surgeons, each of whom have a minimum of 5 years of experience in hip arthroscopy.

At the beginning of the survey, surgeons were asked to provide background information about their training level and their experience in performing arthroscopic procedures. The participants were then asked their perception of the importance of general skills and specific skills for hip arthroscopy on a 5-point Likert scale from least important (given a score of 1) to most important (given a score of 5). There was an open-ended question section that surgeons were able to give comments about any essential techniques needed for hip arthroscopy that were missing in this survey, following which, surgeons were surveyed on the optimal type of simulation for learning hip arthroscopic skills on a 5-point Likert scale from least useful (given a score of 1) to most useful (given a score of 5). The data were collected from June to August 2021.

As this study did not involve treatment of patients or the revelation of any identifiable personal information, ethics approval was waived by the research ethics committee of Chongqing Medical University. Informed consent was obtained from the participants in the online survey before they proceeded to answer questions.

## Analysis and statistics

For analysis, the questions relating to specific skills (24 skills) were further divided into three categories based on the nature of skills [19, 20]: (a) preparation of the patient and instruments; (b) identification of structures and navigation of the arthroscope; (c) instrument handling. For statistical analysis, one-way ANOVA with Tukey pairwise comparisons was performed, and Cronbach’s alpha test was used to evaluate the internal consistency of the questions. The R software (R Foundation) version 4.1.1 was used to complete all statistical analyses.

## Results

A total of 225 arthroscopic professionals responded to the survey. Invalid data and responses received from participants who have no prior experience of hip arthroscopy were excluded. Altogether, 159 responses from surgeons of 130 institutions located across 27 provincial administrative districts of China were finally included in the dataset. Cronbach’s alpha test performed on 33 survey questions (*α* = 0.967) showed that the internal consistency of this survey is “Excellent.” Of the 159 valid responses, 66 responses were from junior specialist surgeons, 68 responses from consultants, and 25 responses from senior consultants. The average number of years of experience for performing arthroscopy was 8.48 (± 4.71) years, while the average number of arthroscopic procedures per year was 267.5 (± 241.1) operations. Details of the demographic information of the participants are presented in Table 1.

**Table 1:** Participants demographics

	Number of participants *n*	Average years of performing arthroscopies mean (± SD)	Average number of arthroscopic operations per year mean (± SD)	Total number of hip arthroscopic operations mean (± SD)
Junior Specialist Surgeons^*a*^	66	5.39 (± 2.80)	164.12 (± 134.96)	178.71 (± 252.79)
Consultants^*b*^	68	9.71 (± 3.86)	276.93 (± 179.22)	377.71 (± 435.65)
Senior Consultants^*c*^	25	13.28 (± 5.25)	514.80 (± 257.71)	1050.48 (± 980.02)
All participants	159	8.48 (± 4.71)	267.50 (± 214.08)	400.89 (± 583.73)
Total number of provincial administrative district in China	34	Surveyed provincial administrative districts	27	Coverage 79.4%

*a* compared with *b*, **; *a* compared with c, ****; *b* compared with *c*, ****

^**^represents *P* < 0.01; ****represent *P* < 0.0001

One-way ANOVA test on participants’ experience level and average number of performed arthroscopic procedures revealed a significant difference (*P* < 0.01). Tukey test for pairwise comparison revealed significant differences between all pairs of experience groups (*P* < 0.01). It is not surprising that senior consultant surgeons tend to perform more arthroscopic procedures than consultants and junior specialist surgeons.

### General skills

The importance of the five general skills from the perspective of professional surgeons as shown in Table 2 is as follows: (1) anatomical knowledge, (2) triangulation/depth perception (using tools to access a specific spot from two portals simultaneously), (3) spatial perception (navigating in a 3D space), (4) manual dexterity, and (5) tactile sensation (Table 2). One-way ANOVA test revealed significant difference across the five different skills (*P* < 0.01).

**Table 2:** Five general surgical skills trainees should possess prior to performing in operating room, rated by surgeons with different levels of experience

	Junior specialist surgeons	Consultants	Senior consultants	All participants
Value	Mean (± SD)	Mean (± SD)	Mean (± SD)	Mean (± SD)
Anatomical knowledge^*a*^	4.65 (± 0.79)	4.71 (± 0.60)	4.52 (± 0.98)	4.65 (± 0.75)
Triangulation/depth perception (using tools to access a specific spot from two portals simultaneously)^*b*^	4.50 (± 0.91)	4.47 (± 0.74)	4.08 (± 1.02)	4.42 (± 0.87)
Spatial perception (navigating in a 3D space)^*c*^	4.35 (± 0.88)	4.46 (± 0.78)	4.00 (± 1.17)	4.34 (± 0.90)
Manual dexterity^*d*^	4.11 (± 1.02)	4.34 (± 0.87)	3.80 (± 1.02)	4.16 (± 0.97)
Tactile sensation^*e*^	3.89 (± 1.17)	3.85 (± 1.13)	3.32 (± 1.54)	3.79 (± 1.24)

*a* compared with *c*, *; *a* compared with *d*, ****; *a* compared with *e*, ****; *b* compared with *e*, ****; *c* compared with *e*, ****; *d* compared with *e*, **

*Represents *P* < 0.05; **Represents *P* < 0.01; ****Represents *P* < 0.0001

Statistical analysis for the average scores of individual questions among surgeons with different experience levels revealed no significant differences, showing that surgeons concur on the importance of these five general skills.

### Specific skills

To establish the trends on the importance of specific surgical skills, the analysis was conducted based on three identified categories, including: (1) identification of structures and navigation of the arthroscope, (2) instrument handling, and (3) preparation of the patient and instruments. The average score of each category was first compared. Next, the mean scores from the surgeons with different levels of experience were compared within each individual category.

As shown in Table 3, surgeons considered skills relating to the identification of structures and navigation as the most crucial skills in the preparation of trainees before they perform the actual surgery. A post hoc Tukey test revealed that skills relating to the preparation of patients and instruments were significantly lower than the other two categories (*P* < 0.05). There were no significant differences found when comparing the scores from surgeons with different levels of experience for each individual category.

**Table 3:** Categories of specific surgical skills important for trainees to possess prior to performing in operating room, rated by surgeons with different levels of experience

	Junior specialist surgeons	Consultants	Senior consultants	All participants
Value	Mean (± SD)	Mean (± SD)	Mean (± SD)	Mean (± SD)
Identification of structures and navigation of the arthroscope^*a*^	4.15 (± 0.81)	4.20 (± 0.68)	4.09 (± 0.78)	4.16 (± 0.76)
Instrument handling^*b*^	4.11 (± 0.81)	4.07 (± 0.65)	4.06 (± 0.76)	4.09 (± 0.74)
Preparation of the patient and instruments^*c*^	3.84 (± 0.86)	3.92 (± 0.77)	3.63 (± 0.96)	3.84 (± 0.85)

*a* compared with *c*, ***; *b* compared with *c*, *

*Represents *P* < 0.05; ***Represents *P* < 0.001

As shown in Table 4, top skills perceived as important for hip arthroscopy include treatment of cam deformity (where the head of the femur does not sit symmetrically on the neck of the femur), establishing the mid-anterior portal under the direct vision of camera on anterior triangle, suturing of the labrum with passing/shutting devices, identification of the insertion needle location, and treatment of pincer deformity (which is an abnormality of the acetabulum). The least important skill for trainees to possess prior to performing actual surgery includes operating room set-up followed by the draping system, removal of tissue with basket forceps and use of arthroscopic blades.

**Table 4:** Specific surgical skills trainees should possess prior to performing in operating room, rated by surgeons with different levels of experience

	Junior specialist surgeons	Consultants	Senior consultants	All participants
Value	Mean (± SD)	Mean (± SD)	Mean (± SD)	Mean (± SD)
Treatment of cam deformity	4.44 (± 0.94)	4.46 (± 0.74)	4.16 (± 1.16)	4.40 (± 0.91)
Establishing the mid-anterior portal under the direct vision of camera on anterior triangle	4.35 (± 0.84)	4.46 (± 0.72)	4.32 (± 0.88)	4.39 (± 0.80)
Suturing the labrum with passing/shutting devices	4.36 (± 0.95)	4.37 (± 0.87)	4.40 (± 0.75)	4.37 (± 0.89)
Identifying the location of insertion needle	4.35 (± 0.88)	4.35 (± 0.82)	4.32 (± 0.93)	4.35 (± 0.86)
Treatment of pincer deformity	4.39 (± 0.94)	4.35 (± 0.76)	4.12 (± 1.18)	4.33 (± 0.92)
Incision and closure of joint capsule	4.23 (± 1.08)	4.34 (± 0.80)	4.28 (± 0.72)	4.28 (0.92)
Assessment of Labrum stability	4.27 (± 0.98)	4.25 (± 0.88)	4.20 (± 0.98)	4.25 (± 0.94)
Insertion of scope to anterolateral portal	4.30 (± 0.89)	4.21(± 0.95)	4.16 (± 1.01)	4.24 (± 0.93)
Patient positioning	4.26 (± 1.09)	4.31 (± 0.81)	3.92 (± 1.29)	4.23 (± 1.03)
Establishing the anterolateral portal under fluoroscopic guidance	4.09 (± 1.07)	4.38 (± 0.86)	4.08 (± 1.13)	4.21 (± 1.01)
Establishing the distal anterolateral portal under the direct vision of camera	4.19 (± 1.01)	4.18 (± 1.06)	3.88 (± 0.99)	4.13 (± 1.03)
Countertraction and air arthrogram (application of opposing traction followed by fluoroscopy to ensure sufficient distraction of the joint during the arthroscopic procedure)	4.06 (± 0.98)	4.24 (± 0.89)	3.92 (± 1.02)	4.11 (± 0.96)
Positioning the camera in the mid-anterior portal to view the blindly placed anterolateral portal	4.12 (± 0.96)	4.15 (± 0.77)	3.96 (± 0.82)	4.11 (± 0.87)
Use of electrocautery	4.12 (± 1.08)	4.10 (± 0.88)	4.08 (± 1.02)	4.11 (± 0.99)
Performing the diagnostic arthroscopy in the central compartment	4.11 (± 0.96)	3.99 (± 0.95)	4.24 (± 0.91)	4.08 (± 0.95)
Precise portal placement	4.11 (0.91)	4.09 (0.98)	3.92 (1.02)	4.07 (0.96)
Shaving of synovium, cartilage, and labrum	3.89 (± 1.06)	3.93 (± 0.88)	4.04 (± 1.04)	3.93 (± 0.98)
Palpation of articular surfaces with probe	3.98 (± 1.08)	3.75 (± 1.08)	3.84 (± 0.97)	3.86 (± 1.07)
Removal of loose bodies with grasping forceps	3.86 (± 1.09)	3.78 (± 0.94)	3.96 (± 1.00)	3.84 (± 1.01)
Establishing the posterolateral portal under the direct vision of camera	3.83 (± 1.07)	3.84 (± 1.11)	3.68 (± 0.88)	3.81 (1.06)
Use of arthroscopic blades	3.74 (± 0.96)	3.66 (± 1.01)	3.88 (± 1.11)	3.73 (± 1.01)
Removal of tissue with basket forceps	3.70 (± 1.04	3.63 (1.03)	3.76 (± 1.14)	3.68 (± 1.05)
Draping system	3.36 (± 1.14)	3.54 (± 1.22)	3.44 (± 1.13)	3.45 (± 1.17)
Operating room setup	3.44 (± 1.15)	3.43 (± 1.13)	2.96 (± 1.15)	3.35 (± 1.15)

### Preferred types of simulation

Surgeons were asked to score the usefulness of four most common types of simulation: (1) cadaveric specimens, (2) virtual reality (VR) simulator, (3) high-fidelity physical models, and (4) low-fidelity bench-top models, in terms of how much it would help them to practice their surgical skills. As presented in Table 5, simulation using cadaveric specimens is the most popular choice, ranking much higher than the other three methods.

**Table 5:** Usefulness of the simulation type in preparing trainees to perform in the operating room, rated by surgeons with different levels of experience

	Junior specialist surgeons	Consultants	Senior consultants	All participants
Value	Mean (± SD)	Mean (± SD)	Mean (± SD)	Mean (± SD)
Simulation using cadaveric specimens^*a*^	4.48 (± 0.89)	4.57 (± 0.77)	4.40 (± 0.75)	4.51 (± 0.82)§‡†
Simulation using high-fidelity physical models^*b*^	3.95 (± 0.99)	3.59 (± 1.02)	3.80 (± 0.80)	3.77 (± 0.99)§※
Simulation using virtual reality simulators^*c*^	3.77 (± 1.06)	3.38 (± 1.11)	3.64 (± 0.93)	3.58 (± 1.08)‡*
Simulation using low-fidelity bench top models^*d*^	3.08 (± 1.09)	2.88 (± 1.13)	3.16 (± 1.25)	3.01 (± 1.14)†※*

*a* compared with *b*, ****; *a* compared with *c*, ****; *a* compared with *d*, ****; *b* compared with *d*, ****; *c* compared with *d*, ****

****Represents *P* < 0.0001

The scores for the 4 simulation methods were significantly different (*P* < 0.01). Tukey test for pairwise comparison of the four simulation methods revealed significant difference among all pairs (*P* < 0.01), except between VR simulator and high-fidelity physical models (*P* = 0.35). The surgeons, regardless of their level of experience, were consistent in their consideration of the importance of each type of simulation. Hence, it is clear that simulation types with higher fidelity are preferred over those with lower fidelity.

For the open-ended question section, participants highlighted that reading skills and interpretation of medical images, time for performing joint traction, and knowledge of the equipment used are items that should be considered in the survey, as these are also important considerations for arthroscopic surgeons to perform the operational procedure successfully.

## Discussion

This present study shows similar trends with earlier studies conducted on Canadian orthopedic professionals [19, 20], although the focus was on arthroscopy of a different joint. Basically, the recognition of the importance of cognitive ability and the method of arthroscopy training for orthopedic professionals in Canada and China converge, even though our present study focused on skills related to hip arthroscopy. The current study has also noted some significant findings that could help to enhance the structure of future training programs for hip arthroscopy.

Hip arthroscopy has proven to be a useful and valuable clinical technique for treating many hip related diseases and symptoms and often with similar or better clinical outcomes in comparison to open surgery [24]. However, this technique has been hindered in the early stage of hip arthroscopy development, by the complex anatomy of the hip joint and related risks [25]. Despite advancements in technology over the years, anatomical issues continue to plague surgeons in the operating room. Surgeons, at all levels of experience, consider sound anatomical knowledge as the most important skill among the five general arthroscopic skills for trainees, that are essential before performing the procedure in operating room.

Based on the survey of the three categories of specific skills, surgeons rated the cognitive aspect of skills that are related to anatomical knowledge and identification of structures higher than motor and technical skills. This could be because cognitive ability have always posed a challenge to endoscopic surgeons who are affected by visuospatial ability [26–29]. During the hip arthroscopic procedure, cognitive skills are required for portal placement since this initial procedure is important for visualization of the joint during the arthroscopic procedure. However, due to the specific anatomical location, portal placement for hip arthroscopy can cause some iatrogenic injuries, such as neurovascular and chondrolabral injuries [30–32], making this technique for hip joint even more demanding as compared to operating on other joints. An earlier report has shown that inexperienced surgeons tend to spend a considerable amount of surgical time on portal placement, which often results in a reduction in time for other treatment procedures while maintaining comparable traction time [33]. This disproportionate allocation of surgical time can deeply impact the overall quality of the surgery and could explain why the participants of this study rated the skills of the portal placement as the most important category for surgical training. The skills in the category of preparation of patient and instruments were rated significantly lower than skills in other two categories. This can be attributed to the fact that the skills in this category are performed prior to the start of the surgical operation. Hence, they are likely to be less important during the surgical procedure than the skills in the other two categories. Moreover, such skills are also easier for trainees to learn and practice outside the operating room.

In this present study, a relatively large number of specific skills have been perceived as important for the surgery. This could explain the reason for the steep and lengthy learning curve for hip arthroscopic surgery. Surgeons would need proficiency in a range of skills before performing the actual surgery in the operating room. It is noted that the top five skills rated by the surgeons are related to FAI. There are reports highlighting that high-impact athletic activities during growth, such as playing soccer, basketball, and ice hockey during adolescence can cause FAI [34–37]. Therefore, a large population ranging from younger to elderly people could all be affected. Hip arthroscopy, the most common surgical technique to address the various types of FAI, has shown optimal clinical outcomes [38, 39]. However, as this surgical procedure is dependent on the acquisition of a good skill set, it requires surgeons to have sufficient experience to make surgical decisions quickly and handle instruments with great dexterity. Therefore, to improve surgeons’ competence in the operating room, it is imperative to enhance the training of FAI related skills in the surgical training program.

In this current study, simulation using cadaveric specimens was rated as the most preferred training method by surgeons. The use of cadaveric specimens have many advantages, including the presence of anatomical structures in-situ, realistic tissue handling and haptic feedback, making this method an excellent approach for medical professionals to practice clinical skills [40]. This training technique would allow trainees to appreciate spatial orientation and handedness when performing clinical procedures, and the combination of visual and haptic feedback can also augment trainees’ memory and help them to recall motor patterns in real clinical settings [40–42]. Using cadaveric specimens is undoubtedly the most ideal method for surgical training as it simulates most closely the scenario in the operating room. Despite the number of benefits associated with using cadaveric simulation, routine application of this training method has met with considerable challenges. This is in part due to cultural, ethical, and legal issues [43, 44], that has limited the availability of cadaveric specimens for medical training. Furthermore, financial considerations, including access to cadavers and costs related to maintaining modern anatomical laboratories, such as establishing a fresh cadaveric facility, are all barriers that could prevent trainees from having sufficient practice [45]. Hence, for routine practice, medical professionals are constantly looking for other practical alternatives.

It is observed that use of a high-fidelity physical model and VR simulator are the most favorable alternatives for medical professionals in practicing their clinical skills [46]. These two kinds of simulation can replicate the clinical operation with great details. Compared to high-fidelity models, a VR simulator tends to have more varied functions that are able to simulate various clinical procedures [11]. Furthermore, the VR simulator gamifies the training experience. Trainees are able to choose modules that are suitable for their experience level, and the simulator can provide immediate feedback on the trainees’ operation [47]. However, there are still contentions broached with regard to the use of VR simulator for clinical training. Since VR-based simulation is costly, the efficiency of training with a VR simulator raises concerns as the educational outcomes do not always justify its high price [48]. Moreover, under certain circumstances, instead of enhancing clinical training, the VR simulator could also possibly increase the cost and difficulty in learning the necessary operative skills [49]. It is therefore plausible that because of these reasons, high-fidelity physical models are more popularly used among surgeons.

High-fidelity physical models can be a full body mannequin or a high-fidelity part-task trainer [10, 50]. This type of simulation has accurate anatomical structures and vivid haptic feedback. In some cases, a physical model combined with electronic components can provide responses required for physical diagnosis or clinical treatment [51]. Since the introduction of medical imaging and 3D printing technologies into simulation design [44, 52, 53], accurate digital models of human structures can be reconstructed and high-fidelity human body replications can be produced by 3D printing in a cost effective way. Using different materials and designs, 3D printed models could be used for a wide range of educational scenarios, from providing anatomical insights to organ transplantation [54, 55]. 3D printed models can simulate general clinical procedures, and customized models that are based on the patients’ medical data to simulate the rarer cases can be developed [56]. Considering that details of bony structure are well presented by medical images, the combination of medical imaging and 3D printing is a promising technique to develop high-fidelity physical models for orthopedic education.

Low-fidelity bench top model seems least favorable by surgeons for the training purposes. This type of simulator usually has simple structures which lack visual and physical representation of the human body and therefore does not allow practice of many clinical procedures. However, this does not mean that low-fidelity simulator is not useful at all for surgical training as under some circumstances, it may offer the same benefits as high-fidelity models [57–60]. For instance, low-fidelity simulation could help trainees to familiarize with instruments and tools that are needed for the surgical procedure and allows trainees to repeatedly practice fundamental skills that are vital for surgery.

## Conclusion

This present study has identified important skills for hip arthroscopic trainees to possess from the perspectives of expert orthopedic surgeons. As cognitive skills have been highlighted as crucial for arthroscopy, medical educators should pay more attention to improve the outcomes of training for cognitive knowledge and skills. The FAI-related skills of hip arthroscopy are also highly emphasized by the surgeons. It would be helpful to design an individual module for the training of such specific skills. Making enhancements to training programs would be useful in helping early career orthopedic surgeons achieve competence in hip arthroscopy, which will result in better clinical outcomes for common diseases of the hip joint. As higher fidelity is favored over lower-fidelity simulation, the development and use of high-fidelity 3D-printed simulator as a viable alternative when cadaveric specimens are not accessible, is recommended to help trainees learn in a more effective way. However, it would also be useful to apply the lower-fidelity simulator in the earlier stage of the training program, before progressing to the use of higher-fidelity models.

## Supplementary Information

Below is the link to the electronic supplementary material.

Supplementary file1 (XLSX 15 KB)

Supplementary file2 (TIF 5570 KB)

## References

[1] Byrd JWT, Jones KS (2011). Arthroscopic management of femoroacetabular impingement: minimum 2-year follow-up. Arthrosc-J Arthrosc Relat Surg.

[2] Ganz R, Parvizi J, Beck M, Leunig M, Nötzli H, Siebenrock KA (2003). Femoroacetabular impingement: a cause for osteoarthritis of the hip. Clin Orthop Relat Res.

[3] Tuijthof GJM, van Sterkenburg MN, Sierevelt IN, van Oldenrijk J, Van Dijk CN, Kerkhoffs GMMJ (2010). First validation of the PASSPORT training environment for arthroscopic skills. Knee Surg Sport Traumatol Arthrosc.

[4] Alvand A, Auplish S, Gill H, Rees J (2011). Innate arthroscopic skills in medical students and variation in learning curves. J Bone Jt Surg.

[5] Carr AJ, Price AJ, Glyn-Jones S, Rees JL (2015). Advances in arthroscopy–indications and therapeutic applications. Nat Rev Rheumatol.

[6] Harris JD (2021). Editorial commentary: virtual reality simulation can help arthroscopic hip preservation surgeons at all levels of training and practice—this is how. Arthrosc-J Arthrosc Relat Surg.

[7] Madan SS, Pai DR (2014). Role of simulation in arthroscopy training. Simul Healthc.

[8] De Boey S, Maes M, Mertens P (2020). Teaching hip surgery to orthopaedic residents: what’s new?. HIP Int.

[9] Bartlett JD, Lawrence JE, Khanduja V (2019). Virtual reality hip arthroscopy simulator demonstrates sufficient face validity. Knee Surg Sport Traumatol Arthrosc.

[10] Phillips L, Cheung JJH, Whelan DB, Murnaghan ML, Chahal J, Theodoropoulos J, Ogilvie-Harris D, Macniven I, Dwyer T (2017). Validation of a dry model for assessing the performance of arthroscopic hip labral repair. Am J Sports Med.

[11] Bauer DE, Wieser K, Aichmair A, Zingg PO, Dora C, Rahm S (2019). Validation of a virtual reality-based hip arthroscopy simulator. Arthrosc-J Arthrosc Relat Surg.

[12] Cook DA, Hatala R, Brydges R, Zendejas B, Szostek JH, Wang AT, Erwin PJ, Hamstra SJ (2011). Technology-enhanced simulation for health professions education. JAMA.

[13] Dawe SR, Windsor JA, Broeders JAJL, Cregan PC, Hewett PJ, Maddern GJ (2014). A systematic review of surgical skills transfer after simulation-based training: laparoscopic cholecystectomy and endoscopy. Ann Surg.

[14] Sonnadara RR, Garbedian S, Safir O, Nousiainen M, Alman B, Ferguson P, Kraemer W, Reznick R (2012). Orthopaedic Boot Camp II: examining the retention rates of an intensive surgical skills course. Surgery.

[15] Mehta N, Chamberlin P, Marx RG, Hidaka C, Ge Y, Nawabi DH, Lyman S (2018). Defining the learning curve for hip arthroscopy: a threshold analysis of the volume-outcomes relationship. Am J Sports Med.

[16] Dumont GD, Gross MM, Cohn RM (2019). The learning curve in hip arthroscopy: effect on total operating room and surgical times. Arthrosc J Arthrosc Relat Surg.

[17] Gomoll AH, O’Toole RV, Czarnecki J, Warner JJP (2007). Surgical experience correlates with performance on a virtual reality simulator for shoulder arthroscopy. Am J Sports Med.

[18] Sutherland LM, Middleton PF, Anthony A, Hamdorf J, Cregan P, Scott D, Maddern GJ (2006). Surgical simulation: a systematic review. Ann Surg.

[19] Safir O, Dubrowski A, Mirsky L, Lin C, Backstein D, Carnahan H (2008). What skills should simulation training in arthroscopy teach residents?. Int J Comput Assist Radiol Surg.

[20] Hui Y, Safir O, Dubrowski A, Carnahan H (2013). What skills should simulation training in arthroscopy teach residents? A focus on resident input. Int J Comput Assist Radiol Surg.

[21] Maier CB, Köppen J, Busse R, Bond C, Elliott R, Bruhn H, Mclaggan D, Archibald D, Ryan M, Skatun D, Heidenreich S, Vlcek F, Zvonickova M, Hodyc D, Svobodová H, Sutton M, Gibson J, McBride A (2018). Task shifting between physicians and nurses in acute care hospitals: cross-sectional study in nine countries. Hum Resour Health.

[22] Lakhani S, Selim OA, Saeed MZ (2021). Arthroscopic simulation: the future of surgical training. JBJS Rev.

[23] Magnussen RA, Granan LP, Dunn WR, Amendola A, Andrish JT, Brophy R, Carey JL, Flanigan D, Huston LJ, Jones M, Kaeding CC, McCarty EC, Marx RG, Matava MJ, Parker RD, Vidal A, Wolcott M, Wolf BR (2009). Cross-cultural comparison of patients undergoing ACL reconstruction in the United States and Norway. Knee Surg Sport Traumatol Arthrosc.

[24] Matsuda DK, Carlisle JC, Arthurs SC, Wierks CH, Philippon MJ (2011). Comparative systematic review of the open dislocation, mini-open, and arthroscopic surgeries for femoroacetabular impingement. Arthrosc-J Arthrosc Relat Surg.

[25] Samim M, Youm T, Burke C, Meislin R, Vigdorchik J, Gyftopoulos S (2018). Hip arthroscopy-MRI correlation and differences for hip anatomy and pathology: what radiologists need to know. Clin Imaging.

[26] Tang B, Hanna GB, Carter F, Adamson GD, Martindale JP, Cuschieri A (2006). Competence assessment of laparoscopic operative and cognitive skills: objective structured clinical examination (OSCE) or observational clinical human reliability assessment (OCHRA). World J Surg.

[27] Angelo RL, Ryu RKN, Pedowitz RA, Beach W, Burns J, Dodds J, Field L, Getelman M, Hobgood R, McIntyre L, Gallagher AG (2015). A proficiency-based progression training curriculum coupled with a model simulator results in the acquisition of a superior arthroscopic bankart skill set. Arthrosc-J Arthrosc Relat Surg.

[28] Anastakis DJ, Hamstra SJ, Matsumoto ED (2000). Visual-spatial abilities in surgical training. Am J Surg.

[29] Wanzel KR, Hamstra SJ, Anastakis DJ, Matsumoto ED, Cusimano MD (2002). Effect of visual-spatial ability on learning of spatially-complex surgical skills. Lancet.

[30] Watson JN, Bohnenkamp F, El-Bitar Y, Moretti V, Domb BG (2014). Variability in locations of hip neurovascular structures and their proximity to hip arthroscopic portals. Arthrosc-J Arthrosc Relat Surg.

[31] Harris JD, McCormick FM, Abrams GD, Gupta AK, Ellis TJ, Bach BR, Bush-Joseph CA, Nho SJ (2013). Complications and reoperations during and after hip arthroscopy: a systematic review of 92 studies and more than 6,000 patients. Arthrosc-J Arthrosc Relat Surg.

[32] Weber AE, Harris JD, Nho SJ (2015). Complications in hip arthroscopy: a systematic review and strategies for prevention. Sports Med Arthrosc.

[33] Kautzner J, Zeman P, Stančák A, Havlas V (2018). Hip arthroscopy learning curve: a prospective single-surgeon study. Int Orthop.

[34] Pun S, Kumar D, Lane NE (2015). Femoroacetabular impingement. Arthritis. J Rheumatol.

[35] Agricola R, Bessems JHJM, Ginai AZ, Heijboer MP, Van Der Heijden RA, Verhaar JAN, Weinans H, Waarsing JH (2012). The development of cam-type deformity in adolescent and young male soccer players. Am J Sports Med.

[36] Siebenrock KA, Kaschka I, Frauchiger L, Werlen S, Schwab JM (2013). Prevalence of cam-type deformity and hip pain in elite ice hockey players before and after the end of growth. Am J Sports Med.

[37] Siebenrock KA, Behning A, Mamisch TC, Schwab JM (2013). Growth plate alteration precedes cam-type deformity in elite basketball players hip. Clin Orthop Relat Res.

[38] Kelly BT, Bedi A, Robertson CM, Dela Torre K, Giveans MR, Larson CM (2012). Alterations in internal rotation and alpha angles are associated with arthroscopic cam decompression in the hip. Am J Sports Med.

[39] Schilders E, Dimitrakopoulou A, Bismil Q, Marchant P, Cooke C (2011). Arthroscopic treatment of labral tears in femoroacetabular impingement: a comparative study of refixation and resection with a minimum two-year follow-up. J Bone Jt Surg Ser B.

[40] Au J, Palmer E, Johnson I, Chehade M (2018). Evaluation of the utility of teaching joint relocations using cadaveric specimens. BMC Med Educ.

[41] Morris D, Hong T, Barbagli F, Chang T, Salisbury K (2007) Haptic feedback enhances force skill learning. In: Proceedings second joint eurohaptics conference and symposium on haptic interfaces for virtual environment and teleoperator systems (WHC’07) (pp. 21-26). IEEE. 10.1109/WHC.2007.65

[42] Feygin D, Keehner M, Tendick F (2002) Haptic guidance: experimental evaluation of a haptic training method for a perceptual motor skill. In: Proceedings 10th symposium on haptic interfaces for virtual environment and teleoperator systems. HAPTICS 2002 (pp 40–47). IEEE. 10.1109/HAPTIC.2002.998939

[43] AbouHashem Y, Dayal M, Savanah S, Štrkalj G (2015). The application of 3D printing in anatomy education. Med Educ Online.

[44] Mcmenamin PG, Quayle MR, Mchenry CR, Adams JW (2014). The production of anatomical teaching resources using three-dimensional (3D) printing technology. Anat Sci Educ.

[45] Raja DS, Sultana B (2011). Potential health hazards for students exposed to formaldehyde in the gross anatomy laboratory. J Environ Health.

[46] Grober ED, Hamstra SJ, Wanzel KR, Reznick RK, Matsumoto ED, Sidhu RS, Jarvi KA (2004). The educational impact of bench model fidelity on the acquisition of technical skill: the use of clinically relevant outcome measures. Ann Surg.

[47] Feldman BH, Ake JM, Geist CE (2007). Virtual reality simulation. Ophthalmology.

[48] Mishra S, Sharma R, Kumar A, Ganatra P, Sabnis RB, Desai MR (2011). Comparative performance of high-fidelity training models for flexible ureteroscopy: are all models effective?. Indian J Urol.

[49] Preece D, Williams SB, Lam R, Weller R (2013). “Let’s get physical”: Advantages of a physical model over 3D computer models and textbooks in learning imaging anatomy. Anat Sci Educ.

[50] McGarry D, Cashin A, Fowler C (2014). Is high fidelity human patient (mannequin) simulation, simulation of learning?. Nurse Educ Today.

[51] Norman G, Dore K, Grierson L (2012). The minimal relationship between simulation fidelity and transfer of learning. Med Educ.

[52] Ackerman MJ (1998). The visible human project. Proc IEEE.

[53] Adams JW, Paxton L, Dawes K, Burlak K, Quayle M, McMenamin PG (2015). 3D printed reproductions of orbital dissections: a novel mode of visualising anatomy for trainees in ophthalmology or optometry. Br J Ophthalmol.

[54] Cai B, Rajendran K, Bay BH, Lee J, Yen CC (2019). The effects of a functional three-dimensional (3D) printed knee joint simulator in improving anatomical spatial knowledge. Anat Sci Educ.

[55] Adams F, Qiu T, Mark A, Fritz B, Kramer L, Schlager D, Wetterauer U, Miernik A, Fischer P (2017). Soft 3D-printed phantom of the human kidney with collecting system. Ann Biomed Eng.

[56] Knox K, Kerber CW, Singel SA, Bailey MJ, Imbesi SG (2005). Rapid prototyping to create vascular replicas from CT scan data: making tools to teach, rehearse, and choose treatment strategies. Catheter Cardiovasc Interv.

[57] Hamstra SJ, Dubrowski A, Backstein D (2006). Teaching technical skills to surgical residents: a survey of empirical research. Clin Orthop Relat Res.

[58] Hamstra SJ, Brydges R, Hatala R, Zendejas B, Cook DA (2014). Reconsidering fidelity in simulation-based training. Acad Med.

[59] Schoenherr JR, Hamstra SJ (2017). Beyond fidelity: Deconstructing the seductive simplicity of fidelity in simulator-based education in the health care professions. Simul Healthc.

[60] Hamstra SJ, Schnurr M, MacLeod A (2021). 12 The natural history of simulation centres: educational support systems or expressions of technology?. Simulations and student learning.

