# Supplementary figures and images for: Training surgical skills on hip arthroscopy by simulation: a survey on surgeon’s perspectives
Source: Int J Comput Assist Radiol Surg. 2022 Jul 13;17(10):1813–21. doi: 10.1007/s11548-022-02708-x (PMC9468038; doi:10.1007/s11548-022-02708-x)

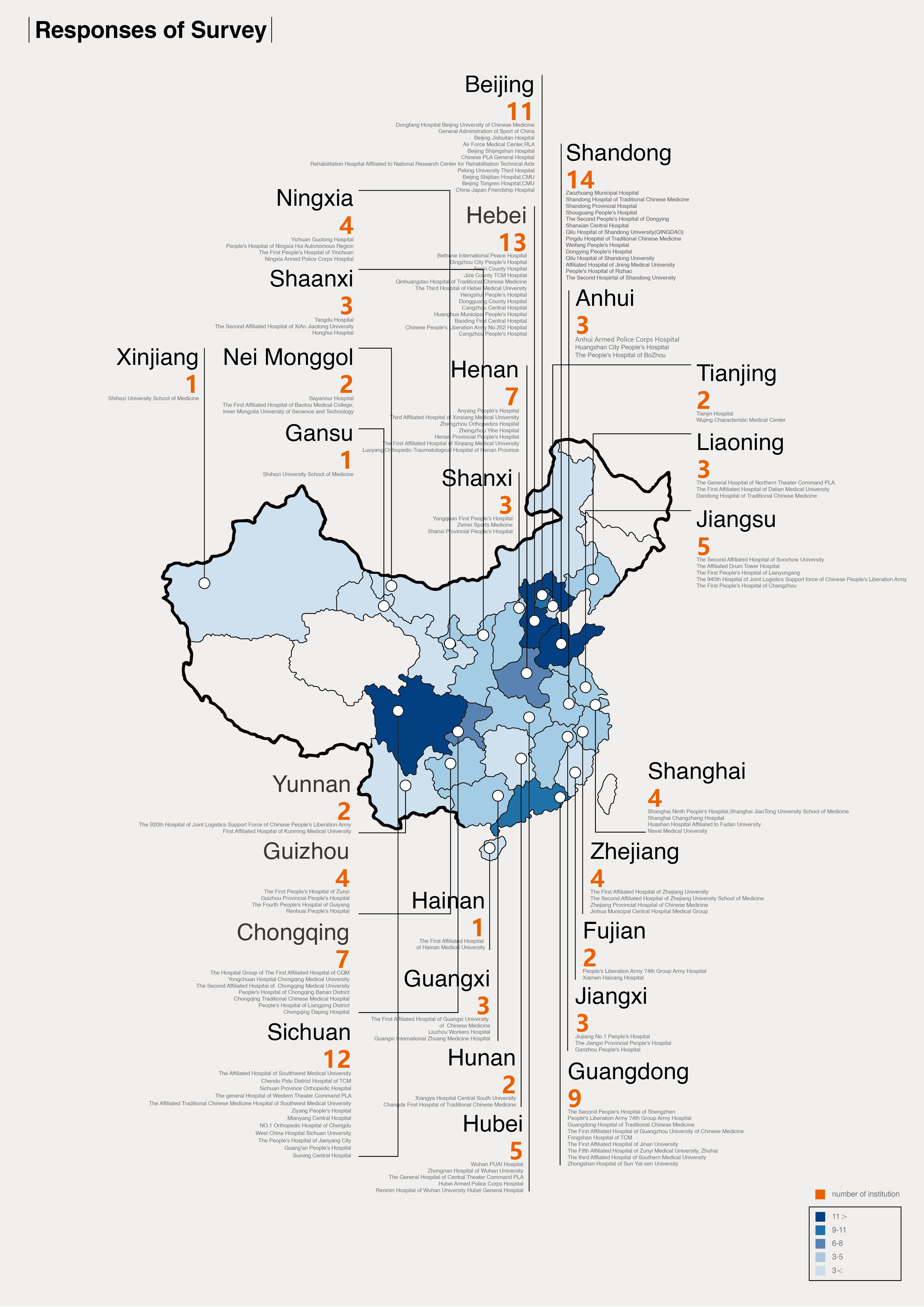

Supplement: Supplementary file 2 — Supplementary file2 (TIF 5570 KB) [file 11548_2022_2708_MOESM2_ESM.tif]
